# Emergency medicine trainees' perceived barriers to training and credentialing in point‐of‐care ultrasound: A cross‐sectional study

**DOI:** 10.1002/ajum.12317

**Published:** 2022-09-23

**Authors:** Tarek Elsayed, Peter J. Snelling, Erin J. Stirling, Stuart Watkins

**Affiliations:** ^1^ Emergency Department Gold Coast Hospital and Health Service Southport 4215 Queensland Australia; ^2^ School of Medicine and Dentistry Griffith University Southport Queensland Australia; ^3^ Faculty of Health Sciences & Medicine Bond University Robina Queensland Australia; ^4^ Child Health Research Centre University of Queensland Queensland Australia; ^5^ Sonography Innovation and Research (Sonar) Group Gold Coast Queensland Australia

**Keywords:** accreditation, bedside ultrasound, credentialing, competency, education, emergency, proficiency, point‐of‐care ultrasound, training

## Abstract

**Introduction:**

Point‐of‐care ultrasound (POCUS) is an important tool in emergency medicine (EM), with the Australasian College for Emergency Medicine (ACEM) recommending core modalities as part of fellowship training. In Australia, acquisition of these skills is certified *via* credentialing but is currently poorly undertaken by EM trainees.

**Methods:**

We performed a cross‐sectional survey of EM trainees across two academic teaching hospitals in Gold Coast, Queensland, between December 2018 and January 2019, to determine the current state of training and perceived barriers to credentialing in POCUS.

**Results:**

Fifty‐two (59%) eligible EM trainees participated. Although credentialing rates (15%) were low amongst respondents, the majority agreed that it was necessary (69%) and should form part of ACEM training (88%). Amongst these trainees, we identified the desire for increased POCUS training and several barriers including time constraints and the credentialing process itself.

**Conclusion:**

Although there is general agreement amongst EM trainees for POCUS credentialing, barriers such as time limitations and technical difficulties were prohibitive for many. We propose the development of an internal structured POCUS training programme within mandatory training time to address these issues.

## Introduction

The use of point‐of‐care ultrasound (POCUS) in emergency medicine (EM) rapidly increased internationally after being first adopted in the 1990s.^1^ It has become a valuable diagnostic and procedural tool that EM clinicians have increasingly incorporated into their scope of practice, to aid in the management of patients in Australia.^1^, ^2^ The effective use of POCUS requires a clinician to identify an indication, acquire images, interpret them and clinically integrate the findings to answer a focussed clinical question. Skipping any of these steps could provide inaccurate information, which could potentially lead to inappropriate decisions.

To ensure standards of use, governing bodies have recommended minimum POCUS training credentialing requirements.^3^ Credentialing is the formal process of verifying a clinician's experience and competence to provide safe, high‐quality POCUS within the workplace. This typically consists of an education programme, performing a requisite number of scans and demonstrating competence with a summative assessment.^2^ In North America, POCUS has been incorporated into EM training programmes, albeit with heterogeneous training methodologies and credentialing processes.^3^, ^4^ The American College of Graduate Medical Education mandates POCUS training during EM training, with a minimum requirement in both teaching hours and scans for accreditation.^5^

In Australia and New Zealand, the Australasian College for Emergency Medicine (ACEM) recommends all EM physicians to be credentialed in five core POCUS modalities: abdominal aorta aneurysm (AAA), extended focussed sonography in trauma (eFAST), focussed echo in life support (FELS), lung, and procedural guidance.^2^ in Australasia, EM training in POCUS is currently based on institutional credentialing processes recommended by ACEM, or by a recognised qualification in POCUS, such as a Certificate in Clinical Performed Ultrasound (CCPU) in the corresponding modules *via* the Australasian Society for Ultrasound in Medicine (ASUM).^6^

However, despite existing pathways for credentialing, there has been relatively low uptake amongst EM trainees. In a survey of Australasian EM fellows and trainees in 2014, only 18% of respondents met ACEM's requirements.^7^ Remarkably, 85% of the respondents believed EM ultrasound training should be compulsory.^7^ This has changed little over the decade; a recent audit of EM doctors in a metropolitan area found that only 33% of participants had their ultrasound skills formally assessed.^8^ This disparity is potentially an indication of the scale of the barriers to POCUS credentialing that exists in the Australian EM training landscape.

The aim of this study was to determine the experience, education and perceived barriers to POCUS training and credentialing amongst EM trainees across two academic hospitals within a health district in South East Queensland, Australia. Secondarily, we use these data to propose potential solutions or strategies to overcome these barriers and increase POCUS credentialing uptake amongst EM trainees within Australia.

## Methods

This was a cross‐sectional study of EM trainees across two hospitals within the Gold Coast Hospital and Health Service (GCHHS), Queensland, Australia, conducted between December 2018 and January 2019. Trainees worked between a 750‐bed tertiary care centre with an annual census of around 110,000 emergency department (ED) presentations, and a large urban care centre with 364 beds and around 62,000 annual ED presentations at the time of the study being conducted. Participation was voluntary, with consent implied when participants completed the survey.

The GCHHS service provides a range of opportunities for training and credentialing in POCUS. There were eight CCPU‐qualified EM sonologists who provide timetabled proctored POCUS scanning, available to all emergency clinicians. An ACEM‐accredited emergency ultrasound special skill training programme is also available for two trainees per year. At least quarterly procedural workshops are held for POCUS‐guided peripheral intravenous cannulation training annually.^9^ A quality assurance process is available to allow trainees to upload images to a secure website to asynchronously receive feedback, whilst forming the basis for their logbook for subsequent credentialing or qualification.

A survey was developed as a structured questionnaire, which included POCUS experience, education and any perceived barriers to POCUS training and credentialing. Participants were eligible for the study if they were registered as an ACEM trainee at the time of distribution. The questionnaire was electronically distributed *via* email, and obtained *via* Survey Monkey® (SurveyMonkey Inc.), an anonymous, Internet‐based electronic survey. Email reminders and a phone call at one month were provided to increase completion rates. Descriptive statistics were used to calculate frequencies and percentages.

### Ethics approval

The Gold Coast Hospital and Health Service Human Research Ethics Committee (EC00160) approved the study (LNR/2018/QGC/47505).

## Results

**Figure 1:**
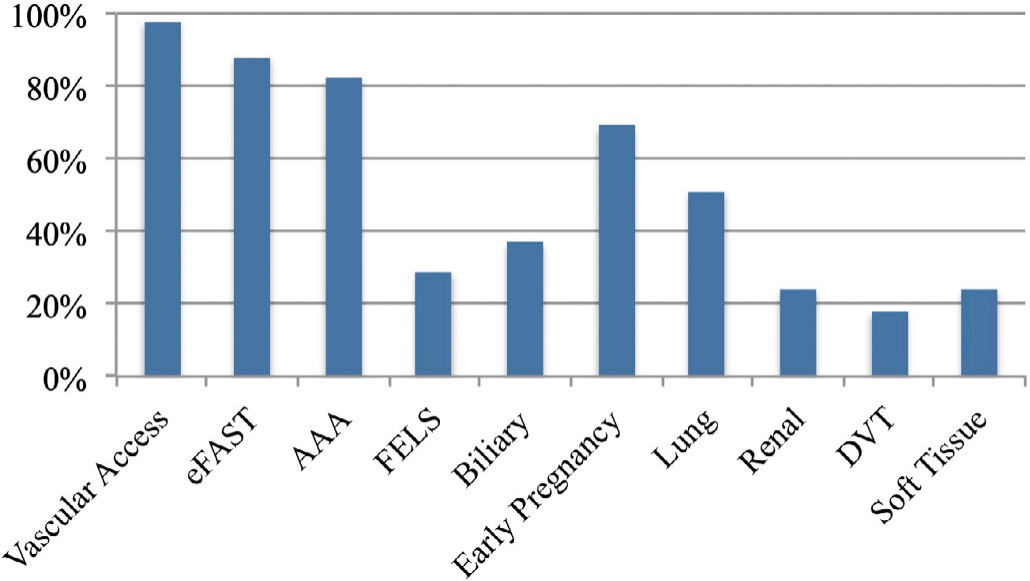
Emergency medicine trainees’ current use of point‐of‐care ultrasound by modality. AAA, abdominal aortic aneurysm; FELS, focussed echocardiography in life support; DVT, deep vein thrombosis; eFAST, extended focussed assessment using sonography in trauma. [Colour figure can be viewed at wileyonlinelibrary.com]

There were 52 (59%) survey responses of 88 eligible EM trainees (Table 1). Most respondents (89%) were in their final 2 years of ACEM advanced training. The majority (85%) had also previously attended at least one accredited POCUS training course, with about a third having completed it within the past year and another third between 1 and 2 years ago. Most respondents were currently undertaking an ED placement (71%). Eight respondents (15%) had achieved the CCPU qualification in at least one module. Vascular access and eFAST were the most utilised POCUS modalities in the clinical setting, with POCUS for deep vein thrombosis being the least utilised modality (Figure 1). Whilst working clinically, 83% of respondents could typically identify at least one person to approach for help with POCUS, whilst 69% could list at least two people. In terms of POCUS self‐education, most respondents used online resources (78%), predominately videos of lectures and tutorials, or teaching from colleagues (78%) (Table 1).

**Table 1:** Survey respondent demographics and POCUS experience

	Respondents *n (%)*
	52 (59)
Year of training	
Provisional	6 (12)
Years 3–4	47 (89)
POCUS course attendance	
1 course	44 (85)
≥2 courses	18 (35)
POCUS course completed	
Within 1 year	15 (29)
Between 1 and 2 years ago	16 (30)
Between 3 and 5 years ago	12 (23)
CCPU qualification – Yes	8 (15)
CCPU qualification modality	
eFAST	7 (13)
FELS	7 (13)
AAA	7 (13)
Biliary	4 (8)
Early pregnancy	3 (6)
Method of POCUS education	
Online videos/lectures	41 (78)
Teaching from colleagues	41 (78)
Websites	31 (60)
Podcasts	23 (44)
Textbooks/journals	10 (19)

AAA, abdominal aortic aneurysm; ACEM, Australasian College for Emergency Medicine; CCPU, Certificate in Clinician Performed Ultrasound; FELS, focussed echocardiography in life support; eFAST, extended focussed assessment using sonography in trauma; POCUS, point‐of‐care ultrasound.

**Figure 2:**
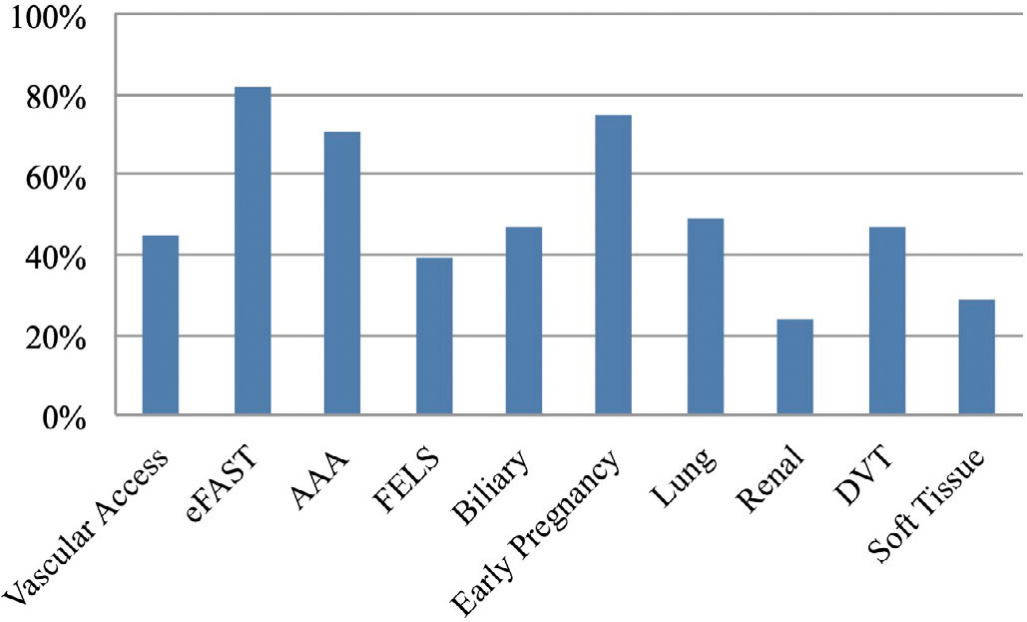
Emergency medicine trainee desired future point‐of‐care ultrasound training by modality. AAA, abdominal aortic aneurysm; DVT, deep vein thrombosis; FELS, focussed echocardiography in life support; eFAST, extended focussed assessment using sonography in trauma. [Colour figure can be viewed at wileyonlinelibrary.com]

Most respondents felt that POCUS training and credentialing should form part of their ACEM fellowship requirements (Table 2). Almost all (88%) indicated this should be incorporated into protected teaching time, with around two‐thirds of respondents indicating that protected POCUS teaching time should be in addition to their current 4‐h ACEM‐mandated education time each week. As a compromise, most trainees indicated interest in substituting their regular education time for ultrasound training. Respondents indicated interest in future training in a wide range of POCUS modalities, with the highest interest in AAA, eFAST and early pregnancy (Figure 2). Overall, over two‐thirds of respondents felt that POCUS credentialing was necessary in some format. However, despite only 15% of respondents reporting formal credentialing, 73% reported ‘occasionally’ or ‘usually’ basing clinical or disposition decisions on their POCUS findings.

**Table 2:** Attitude towards POCUS training and credentialing for ACEM fellowship

	Respondents *n (%)*
‘Should POCUS be a part of ACEM training?’	
As part of 4‐h teaching time – Yes	46 (88)
In addition to 4‐h teaching time – Yes	33 (64)
‘Would you swap regular teaching for POCUS teaching?’	
Yes	23 (44)
Probably	23 (44)
No	5 (10)
‘I do not think that credentialing should be required for ultrasound’	
Strongly disagree	10 (19)
Disagree	26 (50)
Indifferent	11 (21)
Agree	4 (8)
Strongly agree	1 (2)
‘Are you familiar with the ACEM ultrasound credentialing policy?’	
Not at all	8 (15)
A little	11 (21)
A moderate amount	27 (52)
A lot	6 (12)

ACEM, Australasian College for Emergency Medicine; POCUS, point‐of‐care ultrasound.

**Figure 3:**
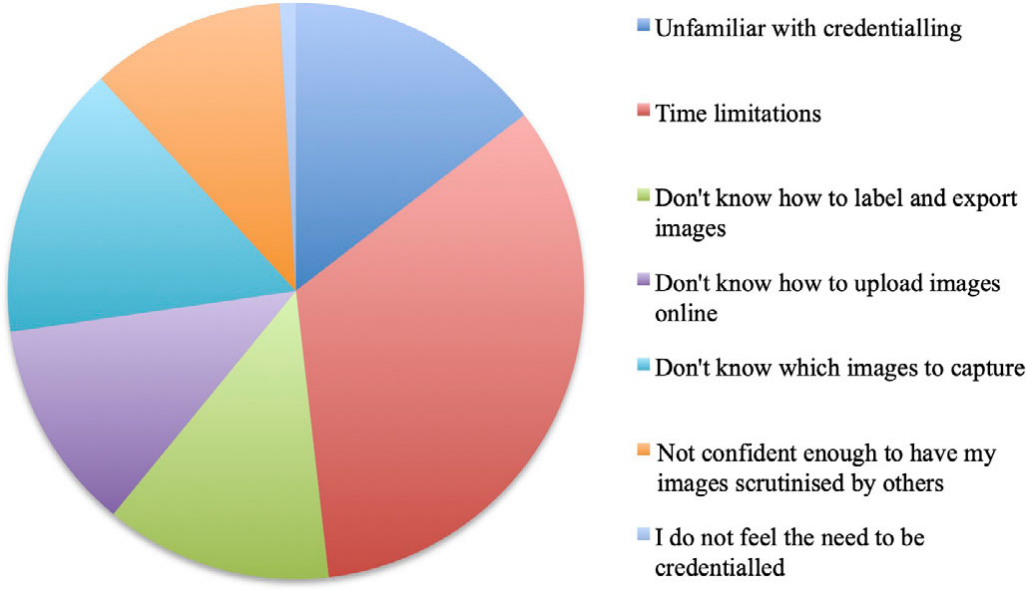
Emergency medicine trainees’ perceived barriers to credentialing. [Colour figure can be viewed at wileyonlinelibrary.com]

A range of barriers to POCUS credentialing was identified, including time limitations, lack of knowledge of the type and number of images to upload for their logbook, lacking confidence for scrutiny of images and unfamiliarity with the credentialing process (Figure 3). Over half of the respondents did not attend proctored scanning sessions in their own time with reasons including lack of awareness of these sessions, being unwilling to attend sessions in unpaid time, and other competing priorities, such as fellowship examination preparation. Over half of respondents felt that the process for credentialing was too onerous, despite 88% perceiving the number of positive scans required for credentialing to be achievable.

## Discussion

This cross‐sectional study explored EM trainee experience and barriers to POCUS training and credentialing within the GCHHS. As the use of POCUS within the ED setting continues to burgeon, appropriate training and credentialing opportunities must be available for EM trainees in Australasia to meet this rising demand. ACEM policy recommends that only credentialed EM clinicians make clinical decisions based on POCUS, making credentialing an important goal for ACEM fellows and trainees to attain.^10^

The requirement of credentialing for the use of POCUS remains controversial.^11^, ^12^ On the one hand, it is seen as a safeguard to ensure appropriate standards and competency are met, but on the other, it is seen as difficult to attain, creating unnecessary ‘red tape’ for many adept users. Only a small proportion (15%) of surveyed EM trainees had successfully completed credentialing in one or more POCUS modalities, in comparison with international EM college trainees who report much higher levels (30–100%).^13^, ^14^ This is explained in part by mandatory ultrasound credentialing, which forms a prerequisite component of their fellowship in many of these countries.^5^ The majority of respondents in our study were in support of mandatory POCUS credentialing, in keeping with a previous Australiasia‐wide study.^7^ If ACEM was to mandate POCUS credentialing as part of EM fellowship training, there would not only be increased uptake in credentialing, but it would also drive the development of increased infrastructure in EDs to accommodate this. Credentialing, despite its limitations, is a formal process of verifying competence in the provision of POCUS and should be feasible regardless of whether it becomes mandatory.

Most trainees indicated that POCUS credentialing should be required for its use, despite many using POCUS whilst uncredentialled. This highlights the current dilemma with ACEM recommending competence *via* credentialing, but trainees nevertheless continue to utilise POCUS in their clinical decision‐making where patient safety takes precedence.^12^ Cobbett *et al*.^15^ recently discussed this tension between formal training and the need for timely access to results, particularly in the context of overnight coverage by trainees who may not be fully credentialed. However, despite the ACEM recommendation, lack of credentialing does not necessarily equate to not being competent in POCUS, but it provides an objective benchmark. An argument can be made that noncredentialled clinicians are potentially exposed to greater scrutiny and wariness from other specialties and raises the possibility of litigation and restrictions on future clinical practice.^16^ Nevertheless, lawsuits in America have been associated with failure to perform POCUS, rather than interpretation errors.^17^ Credentialing, if indeed adopted as the standard for POCUS training, should not only be accessible to EM trainees but should naturally form part of fellowship training as any other routinely performed procedure or skill, for example, endotracheal intubation.

A common barrier identified by the surveyed EM trainees for POCUS credentialing, included poor access to education and training. In terms of POCUS education, most respondents used online resources, in keeping with the advent of FOAMed (free open access medical education) and social media over the past decade.^18^ Although these online resources are easy to access, given their varying quality, and limtied peer review and/or moderation they should not replace standardised training but highlight the need for the availability of ACEM‐endorsed resources (the authors note that ACEM has recently released a range of online training modules). About a quarter of respondents lacked confidence for having their images scrutinised. Educational workshops have been found to be an effective method to increase knowledge and confidence in POCUS.^19^ However, whilst most respondents had attended at least one external POCUS training course in the past 2 years, only a limited number had participated in the POCUS credentialing process. Whilst many Australian EDs have a special skill programme for ultrasound, this is generally limited to 2–4 trainees per year, making it necessary to consider having a vehicle for more widespread training and credentialing. Many Australasian EDs are now developing internal POCUS training courses in line with ACEM guidelines for trainees, although these are not yet widespread and may still require participants to attend in their own time.^20^, ^21^, ^22^

Another identified common barrier to POCUS training and credentialing was time limitation. The issue of time constraints is not unique to Australia, with EM trainees internationally also encountering this barrier to POCUS credentialing.^23^, ^24^, ^25^, ^26^ What is still lacking for EM trainees is the incorporation of POCUS into the standard education programme being delivered. In fact, the majority of respondents indicated that they were happy to undergo more POCUS training in lieu of other educational sessions in their ACEM‐mandated 4 hours of weekly teaching. Furthermore, an educational opportunity cost does not necessarily have to exist if ultrasound is absorbed into clinical cases that incorporate multiple emergency themes. For example, teaching sessions on the management of blunt trauma could include eFAST, or advanced life support could include a section on FELS. Indeed, POCUS has already been included in simulation training sessions that have become a fixture in GCHHS EDs and likely similar centres around Australasia. Given that the primary perceived barrier to credentialing was time limitation, this should be a main consideration in the development of a training programme.

Another identified barrier was problems with the actual credentialing process. Many respondents lacked knowledge of the credentialing process, whilst others found it technically challenging. The lack of knowledge of the credentialing process could be improved by increasing the visibility of ACEM policy requirements and the availability of departmental credentialing processes for a particular institution. Many EM trainees did not know how to perform basic functions, including labelling and exporting images to enable image review, either directly or by uploading them to a secure website. By addressing these basic barriers, many EM trainees would be able to progress with the credentialing process. Additionally, respondents did not perceive the number of positive logbook scans required to be excessive. One Australian study demonstrated that in a tertiary adult ED, a clinician is likely to encounter almost 16 patients with an AAA whilst working 30 clinical hours per week, making the credentialing requirement of three positive AAA patients within a year achievable.^27^ By contrast, the total number of logbook scans required for credentialling was perceived to be onerous by the majority of trainees but may be addressed by providing trainees with inbuilt training time. Interestingly, lack of equipment and availability of staff to approach was not considered an issue for respondents in our study compared with other international studies.^28^, ^29^, ^30^

This study has several limitations. The study was only conducted at two sites within a close geographical area in South East Queensland, Australia, so may not be generalisable to other sites across Australia or New Zealand. Although the response rate of 59% was superior to comparable surveys of EM trainees,^7^, ^23^, ^26^, ^30^ it may be affected by spectrum bias towards those interested or trained in POCUS. Although this study focussed on ACEM trainees, further studies should also identify the barriers for ACEM fellows becoming credentialed in POCUS, which would also improve the training and supervision of trainees.

## Conclusion

Whilst POCUS has become an established skill within the ED, perceived barriers exist with training and credentialing. Most EM trainees surveyed within the GCHHS district believe credentialing is important and show a desire to achieve POCUS credentialing. However, barriers such as time limitation, poor access to training, and technical difficulties with credentialing could be addressed with the development of an internal structured training programme within ACEM mandatory education time. This study has provided baseline information to aid us to develop such a programme, which we propose be integrated into the existing educational curriculum.

## Authorship statement

TE and SW conceived and designed the study. TE conducted the study and data collection. TE, EJS and PJS analysed the data. TE and PJS drafted the manuscript, with input from EJS. SW reviewed the final manuscript. TE takes responsibility for the paper as a whole.

## Funding

No funding information is provided.

## Conflict of Interest

None declared.
